# Classification and Progression Based on CFS-GA and C5.0 Boost Decision Tree of TCM Zheng in Chronic Hepatitis B

**DOI:** 10.1155/2013/695937

**Published:** 2013-01-27

**Authors:** Xiao Yu Chen, Li Zhuang Ma, Na Chu, Min Zhou, Yiyang Hu

**Affiliations:** ^1^Centre of Traditional Chinese Medicine Information Science and Technology, Shanghai University of T.C.M., Cailun Road 1200, Shanghai 201203, China; ^2^Department of Computer Science, Shanghai Jiaotong University, Shanghai 200240, China; ^3^Research Institute of Liver Diseases, Shuguang Hospital, Shanghai 201203, China

## Abstract

Chronic hepatitis B (CHB) is a serious public health problem, and Traditional Chinese Medicine (TCM) plays an important role in the control and treatment for CHB. In the treatment of TCM, zheng discrimination is the most important step. In this paper, an approach based on CFS-GA (Correlation based Feature Selection and Genetic Algorithm) and C5.0 boost decision tree is used for zheng classification and progression in the TCM treatment of CHB. The CFS-GA performs better than the typical method of CFS. By CFS-GA, the acquired attribute subset is classified by C5.0 boost decision tree for TCM zheng classification of CHB, and C5.0 decision tree outperforms two typical decision trees of NBTree and REPTree on CFS-GA, CFS, and nonselection in comparison. Based on the critical indicators from C5.0 decision tree, important lab indicators in zheng progression are obtained by the method of stepwise discriminant analysis for expressing TCM zhengs in CHB, and alterations of the important indicators are also analyzed in zheng progression. In conclusion, all the three decision trees perform better on CFS-GA than on CFS and nonselection, and C5.0 decision tree outperforms the two typical decision trees both on attribute selection and nonselection.

## 1. Introduction

With a history of 2000 to 3000 years, Traditional Chinese Medicine (TCM) has formed a unique system to diagnose and cure illness. In TCM, the treatment of illness is based primarily on the diagnosis and differentiation of syndromes [1]. For the TCM theory of “treatment based on zheng differentiation,” a different “zheng” stands for a different syndrome of the disease. A syndrome enables the doctor to determine the development and the location of the disease [2]. Syndrome differentiation is the method of recognizing and diagnosing diseases or body imbalances by analyzing clinical information based on TCM theories and the doctor's experience [3]. TCM has formed a systematized methodology of diagnosis and treatment based on the rich practical knowledge and experience of Chinese people in struggling against diseases. According to the clinical information, practitioners of TCM will perform diagnosis and draw conclusions about the patient's pathological conditions using the term of syndrome (i.e., zheng in Chinese). Chronic hepatitis B (CHB) is a worldwide public health problem for human beings, and TCM shows a positive significance in its treatment and control, but the current standardization of TCM zhengs for CHB has not made a breakthrough. In 1992 and 2002 [4, 5], the Medicine Committee for Liver Disease of the Chinese Medicine Institute and the State Drug Administration formulated the trial viral hepatitis TCM standards and the Chinese medical clinical research guidelines for CHB, respectively, but it still lacks an objective standard for the specific TCM syndromes of CHB. Physicians cannot apply the prescriptive methodology in a professional standard until or unless they have mastered the zheng differentiation process. This limits the clinical efficacy and wide acceptance of TCM to some extent. Therefore, exploring the nature of the TCM zhengs in CHB and the relation between symptoms or lab indicators and a zheng becomes a necessary task for the establishment of the evaluation system for CHB. Modern medical datasets are complex and composed of a great many attributes (symptoms and lab indicators), so they tend to be analyzed through statistics, data mining and other quantitative analysis methods [6–8]. In the attempt to achieve an effective and objective standard of zheng diagnosis, researchers have used the data mining approach to construct the classifier for the TCM dataset, and some research efforts for zheng classification of TCM have been acquired. Xu et al. [9–11] employed CCD devices to acquire tongue images, and analyze the tongue color, shape, texture, moisture, and so on. Then he established a tongue diagnosis system. Li et al. [12, 13] defined a five-color classification scale in complexion diagnosis based on TCM theories and developed an automatic analysis system for assistance in facial complexion-based diagnosis, which standardizes the facial complexion-based diagnosis and avoids depending on the physician's experience and environment. Some scholars [14, 15] have also developed various forms of pulse analysis instruments and studied single and many point sensors for obtaining a mechanical parameter which is helpful for the quantitative analysis of the pulse signals in TCM.

In this paper, the research is based on the clinical investigation of CHB samples. According to CHB lab indicators, it aims to establish the classification approach and explore the relevant indicators in the progression of the TCM zhengs for Damp Heat in the Liver and Gallbladder, Liver Qi Stagnation and Spleen Deficiency, and Yin Deficiency of Liver and Kidney.

## 2. Material and Methods

### 2.1. Material

The CHB dataset of this research is supported by the National Science and Technology Major Project of China (2012ZX10005001-004). The clinical research has been approved by the IRB (Institutional Review Board) of Shuguang Hospital affiliated with Shanghai University of TCM, and all the patients have signed and provided a written informed consent. The dataset includes 550 records of 217 cases originating from CHB patients of Shanghai Shuguang Hospital, Ningbo Infectious Disease Hospital, and the Sixth People's Hospital of Shaoxing. The data records date from November 2009 to April 2010, including 168 male patients (77.34%) and 49 female patients (22.66%). The average age of the patients is (36.94 ± 10.76) years old, the average age of male patients is (36.49 ± 10.23) years old, and the average age of female patients is (38.26 ± 2.13) years old.

Each case has two or three records of zheng differentiation in the dataset, and the time interval of the records of a case is four weeks. 550 records in the dataset are differentiated by physicians and three TCM experts, respectively. The physicians and experts all agreed on the zheng classification for each of these records. These records are divided into three zhengs in TCM: 306 records are the Damp Heat in the Liver and Gallbladder Zheng (A zheng), 155 records are the Liver Qi Stagnation and Spleen Deficiency Zheng (B zheng), and 89 records are the Yin Deficiency of Liver and Kidney Zheng (C zheng), and the values of “A, B, C” correspond to “1, 2, 3” respectively. The 217 cases in the CHB dataset are chosen as the studied material for the changes of zheng type in their corresponding records. Items in the dataset include 83 lab indicators, such as routine blood tests, urine and liver function indicators (including ALT, AST, GGT, AKP, TBIL, PT, APTT, albumin, and globulin, etc.), viral indicators (HBsAg, HBsAb, HBeAg, HBeAb, HBcAb, HBV-DNA, etc.), immune indicators (CD3+, CD4+, CD8+, etc.), renal function (Cr, BUN), blood glucose, and lipids (TG, TC, etc.).

In the dataset, each record has 84 attributes, including 83 clinical lab indicators of CHB and 1 TCM zheng label, and the attributes are encoded by the following rules.

- In the dataset, some lab indicators are encoded with binary values (0, 1), with 0 representing negative and 1 for positive; some are encoded by the four-value ordinal scales measured by the level degree, with 0 representing the normal level, 1 for the slight level, 2 for the medium level, and 3 for the serious level; the others are continuous values.
- The missing values of cases in this dataset are replaced by the mean values of the corresponding attributes. Missing values of the 83 attributes are less than 5%.

### 2.2. Diagnosis, Inclusion, and Exclusion Criteria for CHB and TCM Zhengs

The diagnosis criteria for CHB are referred to in “Prevention and treatment programs of viral hepatitis” [16] issued by the Chinese Liver Disease Association and the Society of Infectious Diseases. It includes the following:

1. cases that are hepatitis B with positive HBeAg, HBsAg and HBV-DNA, negative HBeAb, continuous or repeated elevation in ALT, or hepatitis alterations in liver histological examination,
2. cases that are hepatitis B with negative HBeAg, positive HBsAg and HBV-DNA, continuous or repeated elevation in ALT, or hepatitis alterations in liver histological examination.

The inclusion criteria for CHB include the following:

1. it conforms to the diagnosis criteria of chronic hepatitis B,
2. the indicators of ALT or GGT are abnormal,
3. the age ranges from 18 to 65 years old.

The exclusion criteria include the following:

1. cases of hepatitis B combined with another hepatitis virus,
2. cases of chronic severe hepatitis and cirrhosis,
3. cases of pregnant or lactating women,
4. people who cannot express their feelings clearly.

The category criteria (i.e., the inclusion criteria for TCM zheng) for the three zhengs in CHB are referred to in “TCM syndrome differentiation standards of viral hepatitis (Trial)” [17] issued by the Internal Medicine Department Committee of Liver Disease in Traditional Chinese Medicine Association. The inclusion criteria are based on clinical features of the three zhengs, and the cases that cannot be diagnosed as one of the three TCM zhengs will be excluded.

(A)* Damp Heat in the Liver and Gallbladder.* The major features are (1) yellow skin and eyes, and (2) a yellow, greasy tongue coat.

The minor features are (1) nausea and anorexia, (2) lateral thorax distension and epigastria depression, and (3) yellow urine.

The inclusion criteria for Damp Heat in the Liver and Gallbladder are as follows:

1. cases that have all the major features,
2. cases that have the major feature (1) and two minor features,
3. cases that have the major feature (2) and the minor features (1) and (2).

(B) *Liver Qi Stagnation and Spleen Deficiency*. The major features are (1) distending pain of lateral thorax and (2) abdominal distension and loose stools.

The minor features are (1) chest distress and depression, (2) lassitude and fatigue, and (3) a pink and tooth-marked tongue.

The inclusion criteria for Liver Qi Stagnation and Spleen Deficiency include the following:

1. cases that have all the major features,
2. cases that have the major feature (1) and the minor features (2) and (3),
3. cases that have the major feature (2) and the minor feature (1).

(C)* Yin Deficiency of Liver and Kidney Zheng*. The major features are (1) dizziness and dry eyes, (2) soreness of loins and soft knees, and (3) a red and dry tongue.

The minor features are (1) vexing heat in the chest, palms and soles, (2) insomnia, (3) dull pain of lateral thorax, aggravated by labor, and (4) thready and rapid pulse. 

The inclusion criteria for Yin Deficiency of Liver and Kidney include the following:

1. cases that have all the three major features,
2. cases that have two major features and two minor features,
3. cases that have one major feature and three minor features,
4. cases that have all the four minor features.

### 2.3. Methods

**Figure 1:**
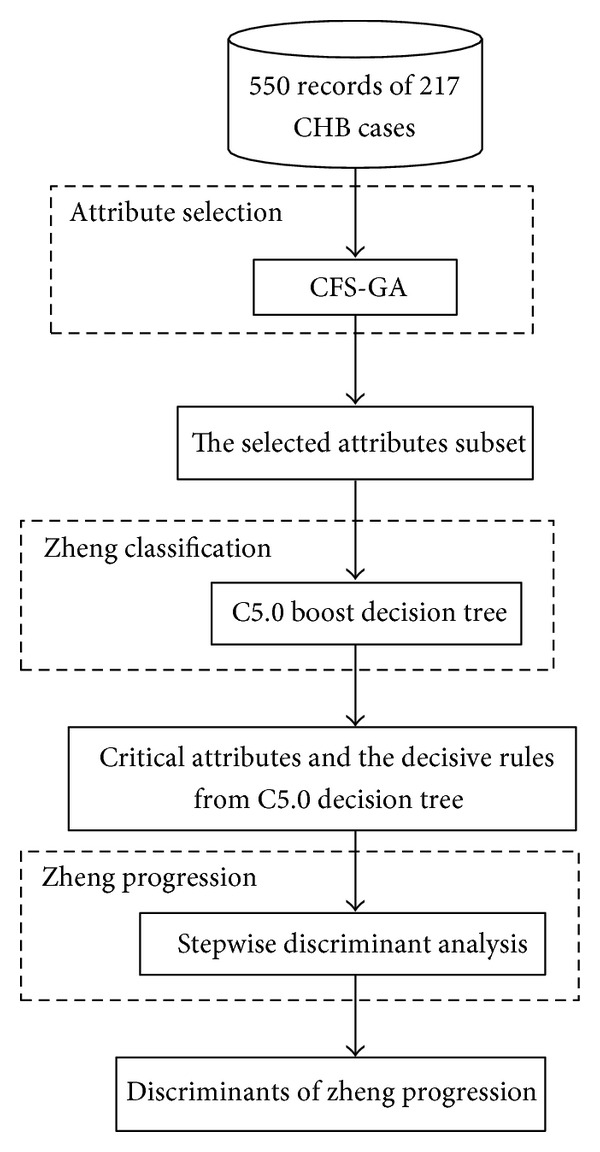
Logic process of TCM zheng classification and progression in CHB.

In this paper, the logic process of this research can be generalized as a three-part architecture including CFS-GA algorithm for attribute selection, C5.0 boost decision tree for classification, and stepwise discriminant analysis for progression, and it is shown in Figure 1.

#### 2.3.1. Attribute Selection of CFS-GA

Modern medical datasets inevitably contain plenty of redundant and irrelevant attributes. Redundant and irrelevant attributes can lower the efficacy of data mining algorithms, causing uninterpretable results, that is, the Hughes phenomenon [18]. The appropriate subset of attributes can yield an accurate and interpretable result for focusing on the significant attributes objectively in zheng differentiation. Therefore, attribute selection is a very important preprocessing step in data mining and analyzing methods. For overcoming the problem of the Hughes phenomenon, attribute subset selection has been used for data reduction in areas characterized by dimensionality due to the large number of available attributes [19]. The CFS-GA algorithm is employed as the attribute selection part in this architecture. CFS (Correlation-based Feature Selection) is a classical filtered algorithm of attribute selection; in this algorithm, the heuristic evaluation for a single feature corresponding to each category label is used to obtain the final feature subset, and the assessment method of CFS is as follows:

(1) $$\begin{array}{c} Ms=\frac{k\bar{r_{cf}}}{\sqrt{k+k\left(k-1\right)+\bar{r_{ff}}}}. \end{array}$$

In (1), *Ms* is the evaluation for an attribute subset *s* including *k* attribute items, ${\bar{r}}_{cf}$ is the mean correlation degree between attributes and the category label, and ${\bar{r}}_{ff}$ is the mean correlation degree among attributes. And the evaluation of CFS is a method of correlation based on attribute subsets. A bigger ${\bar{r}}_{cf}$ or smaller ${\bar{r}}_{ff}$ in acquired subsets by the method produce a higher evaluation value, and in CFS, the correlation degree among attributes is calculated by information gain, and the formula of information gain is shown below. *Y* is the category attribute, *y* is any possible value of *Y*, the entropy of *Y* is shown in (2), and for an attribute *X*, entropy of category attribute *Y* under the condition of *X* is in (3). one has

(2) H(Y)=−∑y∈Yp(y)log2(p(y)),

(3) H(Y ∣ X)=−∑x∈Xp(x)∑y∈Yp(y ∣ x)log2(p(y ∣ x)).

The difference of *H*(*Y*)−*H*(*Y|X*) (i.e., the entropy reduction of attribute *Y*) can reflect the information amount provided by attribute *X* to attribute *Y*, and a bigger difference means a higher correlation degree between *X* and *Y*. Information gain is a symmetrical evaluation method; it tends to select the attributes with more values. Therefore, it is necessary to normalize information gain to [0, 1] for keeping equivalent comparison effect among attributes, and (4), below, shows the calculating formula. one has

(4) $$\begin{array}{c} U_{XY}=2.0\times \frac{H\left(Y\right)-H\left(Y\;|\;X\right)}{H\left(Y\right)+H\left(X\right)}. \end{array}$$

As a filtering algorithm, CFS evaluates the correlation between attributes and category label, and the redundancy degree among attributes [20]. Although the algorithm performs well in dimension reduction, it cannot approach a global optimum result. The Genetic Algorithm (GA) is a wrapping algorithm in dimension reduction for its global search capability [21–23]. In this paper, CFS and GA are combined to make the CFS-GA algorithm, and this algorithm evaluates new individuals of GA through the correlation degree in CFS as the fitness function of GA. The design of the CFS-GA algorithm mainly includes four parts: coding scheme, selection operator, crossover operator, and mutation operator.

In the coding scheme, each entity is encoded with classical binary code. The method of roulette wheel is employed for selection operator. For the crossover operator, single-point crossover is used to produce new individuals by swapping the cross-point part through the crossover points. And basic bit mutation is used in binary encoding for the mutation operator, from 0 to 1, or from 1 to 0.

In the selection of the crossover rate and mutation rate, for producing more new individuals and avoiding causing too much damage to the better attribute subset, the crossover rate range is from 0.40 to 0.99 and the mutation rate is from 0.0001 to 0.1 commonly. The description of CFS-GA algorithm is shown in Algorithm 1.

#### 2.3.2. Classification of C5.0 Boost Decision Tree

As a classification algorithm, decision trees are always praised for comprehensibility of their knowledge representation and inference procedures [24]. Decision trees have been applied widely in classification, prediction, rules extraction, and other areas to solve the key issues of data classification as an indispensable technology in data mining; they are particularly suitable for the complex principles, processes, and relations found in TCM, and some decision tree algorithms are more representative in broader applications, such as ID3 [25] and C4.5 [26] based on information entropy. 

The fundamental idea of a decision tree is to find the decisive attributes through a top-down recursive method and depending on proper values to determine the nodes down from the branches and acquire conclusions in the leaf nodes of the tree. The training set needs to be partitioned recursively, until all records of each subset belong to one class, or the predominant majority of each subset belong to one class. So each path from the root to a leaf node corresponds to a conjunctive rule, and the whole decision tree corresponds to a group of extracted rules, and the relevant algorithm is shown in Algorithm 2. 

Considering the characteristics of the CHB dataset, classification of zheng is based on the method of C5.0 boost decision tree, and it is fit for continuous or nominal attributes in datasets. As the commercial version of C4.5, C5.0 boost decision tree improves the aspects of generating rules and algorithm precision to achieve more accurate generation rules, faster speed, and lower error rate, it is more suitable for classification of large data sets [27].

#### 2.3.3. TCM Zheng Progression of CHB

The zheng progression part of this logic process architecture is based on discriminant analysis. Discriminant analysis [28] is a method based on an available number of samples classified by a number of clear indicators gained through observation, and it provides a discriminant function based on the indicators for classification. Then classify new samples into two types A and B according to the discriminant function to make the lowest mistake classification rate. Methods of discriminant analysis can be divided into the following: Fisher, maximum likelihood, Bayes formula, and gradual selection or full model discriminant analysis [28]. Based on the above methods, in this paper, we use stepwise Fisher's discriminant analysis, and (5) shows the expression below:

(5) $$\begin{array}{c} Z_{}=C_{1}X_{1}+C_{2}X_{2}+\cdots +C_{m}X_{m}. \end{array}$$

In (5), *C*_*i*_ is the coefficient of the equation, *X*_*i*_ is the indicator, and samples are divided into two groups through calculating the values of this function. If the values of *Z* are bigger than the cutoff value *ZC* for classification, samples are classified as type A; if the values of *Z* are smaller than *ZC*, samples are classified as type B; and if the values of *Z* are equal to *ZC*, samples cannot be classified as A or B. The equation of *ZC* is listed as follows:

(6) $$\begin{array}{c} ZC=\frac{\bar{Z_{\text{A}}}+\bar{Z_{\text{B}}}}{2}. \end{array}$$

## 3. Results

### 3.1. Attribute Selection of CFS-GA

The CFS-GA algorithm parameters are set up as follows: the population size and the number of generations are 20, the probabilities of crossover and mutation are 0.6 and 0.033, respectively. 22 lab indicators are selected through CFS-GA algorithm, and they are listed in Table 1.

### 3.2. TCM Zheng Classification of CHB

Accuracy is an evaluation index for classification algorithms. It is calculated as the percentage of the correctly classified samples over all the samples. The C5.0 boost decision tree is used to obtain 13 critical lab indicators in Table 2, and the decision tree induction leads to 12 decisive rules in Algorithm 3 for TCM zheng classification of CHB. An accuracy of 73.82% has been approached in zheng classification of 550 CHB data records.

### 3.3. Comparison

CFS-GA algorithm in attribute selection is compared with CFS and nonattribute selection on three classification methods of the NBTree [29], REPTree [30], and C5.0 boost decision tree on this CHB dataset in classification through tenfold cross-validation.  Table 3 shows a comparison of REPTree, NBTree, and C5.0 boost decision tree, for the three methods on attribute subsets of CFS, CFS-GA, and nonattribute selection. This table proves that the CFS-GA algorithm generally outperforms CFS and nonattribute selection on the three classification methods, and the combination of CFS-GA and C5.0 boost decision tree performs better than the others.

### 3.4. TCM Zheng Progression of CHB

For obtaining the key indicators in the progression of the TCM zheng for CHB, we classify 550 records of 217 CHB patients based on the data view of 13 decisive lab indicators from the decision tree through stepwise Fisher's discriminant analysis. The entry and removal values of F probability in discriminant analysis are set up as 0.05 and 0.10 individually. According to the TCM theory, CHB mainly includes three pathology syndromes in TCM: Damp Heat in the Liver and Gallbladder (A zheng), Liver Qi Stagnation and Spleen Deficiency (B zheng), and Yin Deficiency of Liver and Kidney (C zheng); A and B zheng are the former stages, and C zheng is the latter stage. That means the progression of CHB in TCM develops from A or B zheng to C zheng. In order to study the progression among TCM zhengs, we divide the three-category dataset into two-category datasets, B versus C and A versus C for precise discrimination between syndromes.

From this approach, three relevant lab indicators in zheng progression of CHB are filtered out; they are HBsAg, Eosinophil, and LDL-C, respectively. These indicators reveal a relatively close relationship in Algorithm 4, and on the three indicators; 57.4% and 55.8% of the records are discriminated correctly.

### 3.5. Differences of Relevant Lab Indicators in TCM Zheng Progression

In Section 3.4, we have obtained three relevant indicators in zheng alteration in CHB. To observe the differences of the indicators in zheng alteration, we use all the 190 records of all the 70 cases of C zheng, including 73 records of A zheng, 40 records of B zheng, and 77 records of C zheng. The differences of LDL-C, HBsAg, and Eosinophil are shown in Table 4.

## 4. Discussion

### 4.1. Critical Indicators from CFS-GA and C5.0 Decision Tree

As stated earlier, redundant and irrelevant attributes of datasets will lower the efficacy and performance of data mining algorithms and cause incomprehensible results, so attribute selection is a very important step in the preprocess of sample classification. 

In Table 1, 22 lab indicators related to the three TCM zhengs in CHB are filtered out through CFS-GA algorithm. Based on these indicators, records of the CHB dataset are classified by the C5.0 boost decision tree. The decision tree induction is generalized as 12 critical lab indictors in Table 2 and the decisive rules of Algorithm 3. From Tables 1 and 2, the correlations between some of the indicators with TCM zhengs of A, B, and C in CHB have been proved through medical researches in recent years, such as TBIL, HBsAg, HBcAb-IgM, IgG, albumin, and gamma globulin.

For instance, Zhang et al. [31] detected the liver function indicators of 119 chronic hepatitis B cases and found that TBIL was higher in Damp Heat in the Liver and Gallbladder than in the zhengs of Liver Qi Stagnation and Spleen Deficiency and Yin Deficiency of Liver and Kidney (*P* < 0.05), and it was verified by Jiang et al. [32] and Ma and Liu [33] in the later researches. Zhang et al. [34] observed the expression of HBsAg in liver tissues of 69 chronic hepatitis B cases. The results showed significant differences in the three zhengs of Damp Heat in the Liver and Gallbladder, Liver Qi Stagnation and Spleen Deficiency, and Yin Deficiency of Liver and Kidney (*P* < 0.05). Yu et al. [35] and Zhang et al. [36] found the average titers of HBsAg in Damp Heat zheng and Yin Deficiency of Liver and Kidney are higher than those of Liver Qi Stagnation and Spleen Deficiency (*P* < 0.05). Zhang et al. [36] also discovered positive HBc-IgM with HBsAg together in Liver Qi Stagnation and Spleen Deficiency. In the research of Chang et al. [37], the index of albumin in Yin Deficiency of Liver and Kidney is obviously lower than those in other TCM zhengs (*P* < 0.05), with a rise in Gamma globulin. This was partly verified by the research of Shi [38]. And Shi [38] also discovered that the IgG index in Damp Heat in the Liver and Gallbladder is higher than that in the zhengs of Liver Qi Stagnation and Spleen Deficiency and Yin Deficiency of Liver and Kidney with a significant difference (*P* < 0.05).

### 4.2. Comparison of Classification Results

From Table 3, attribute selection of CFS-GA or CFS algorithm always performs better than nonattribute selection in the three classification algorithms. This proves that attribute selection is an important step before classification and it can improve the accuracy of classification algorithms. Compared to CFS, the CFS-GA algorithm can produce a proper attribute dimension reduction on the CHB dataset, so CFS-GA totally performs better than CFS between the two attribute selection methods. Attribute selection can improve accuracies of classification methods and attribute dimensions. However, a low attribute dimension of data records indicates less information, and it can also influence the efficacy or accuracy of classification methods. In Table 3, although CFS can reduce the attribute dimension of the dataset, CFS has a lower classification accuracy than nonattribute selection for a low attribute dimension in the C5.0 decision tree.

### 4.3. Relevant Indicators in the TCM Zheng Progression of CHB

In the progression of CHB, 550 data records originate from 217 patients, so the data records are classified to reflect TCM zheng progression with the method of stepwise Fisher's discriminant analysis between TCM zhengs in CHB, and three related lab indicators of HBsAg, Eosinophil, and LDL-C are filtered out. Among them, HBsAg and Eosinophil are related to the progression from Liver Qi Stagnation and Spleen Deficiency (B zheng) to Yin Deficiency of Liver and Kidney (C zheng) to some extent, and LDL-C is relatively close to the progression of Damp Heat in the Liver and Gallbladder (A zheng) to C zheng, responding to the three indicators. Classification expressions for the three TCM zhengs are obtained for the progression of B to C zheng and A to C zheng in Algorithm 4, and according to the related researches [33, 34], HBsAg has been proved to be relevant in the three TCM zhengs of CHB.

From Table 4, we can see that the mean value of HBsAg has a significant difference between B zheng and C zheng (*P < 0.05), the mean value of LDL-C has a significant difference between A zheng and C zheng (**P < 0.05), and there is no significant difference of Eosinophil between B zheng and C zheng. It shows that among the three lab indicators in Section 3.4, HBsAg and LDL-C are altered, respectively, in the progressions from B to C and A to C, and the differences of HBsAg in B zheng and C zheng were verified by the former researches [34–36].

## 5. Conclusions

The present study has classified clinical lab indicator records of TCM zheng in CHB through the attribute subset from CFS-GA algorithm and C5.0 boost decision tree, and stepwise discriminant analysis is used for TCM zheng progression of CHB. It reveals three lab indicators in the progression of the three TCM zhengs in CHB. Among the indicators, there are alterations of HBsAg and LDL-C in the progression of TCM zheng. HBsAg in Liver Qi Stagnation and Spleen Deficiency has a significant difference from that in Yin Deficiency of Liver and Kidney, and there is a significant difference of LDL-C between Damp Heat in the Liver and Gallbladder zheng and Yin Deficiency of Liver and Kidney zheng. The proposed approach compares the two decision tree algorithms on attribute subsets from CFS, CFS-GA, and nonselection, respectively. In the comparison, CFS-GA performs better than CFS and nonselection in all the three decision tree methods, and C5.0 boost decision tree performs better than REPTree and NBTree in classification on CFS, CFS-GA, and nonselection. In future research, we will devote ourselves to optimizing the proposed approach and constructing analysis based on more sample sets.

## Figures and Tables

**Algorithm 1:**
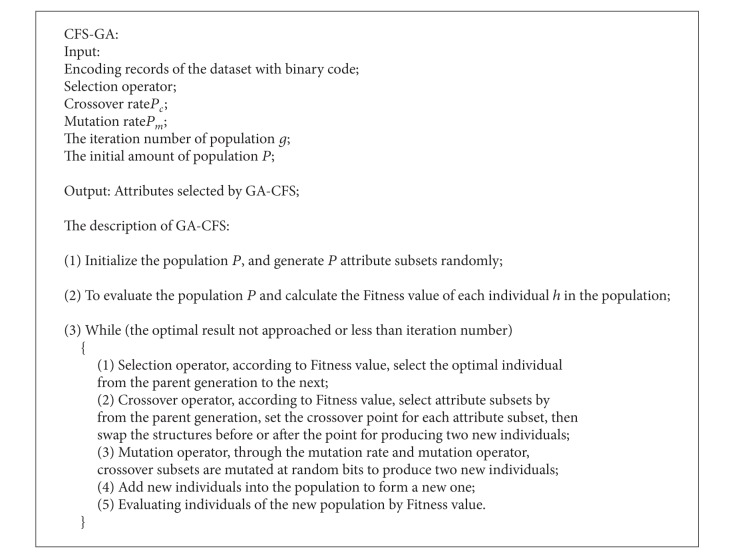
The description of CFS-GA algorithm.

**Algorithm 2:**
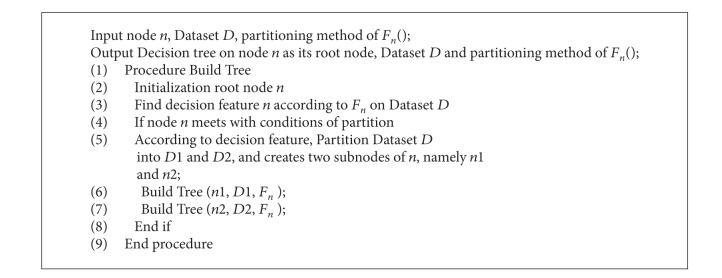
The general description of decision tree.

**Algorithm 3:**
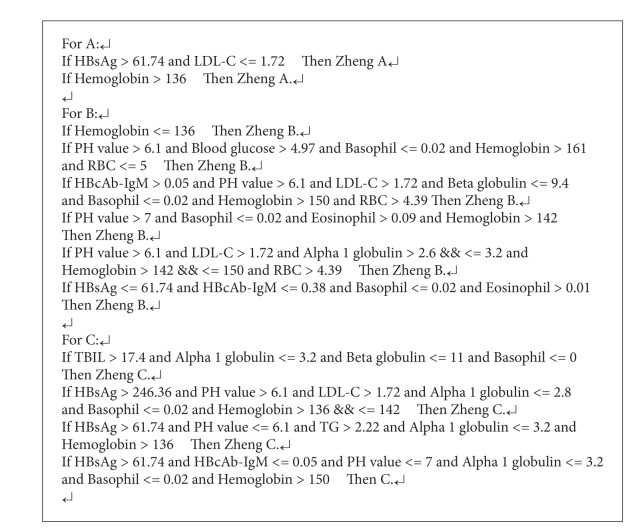
Rules of C5.0 decision tree induction.

**Algorithm 4:**
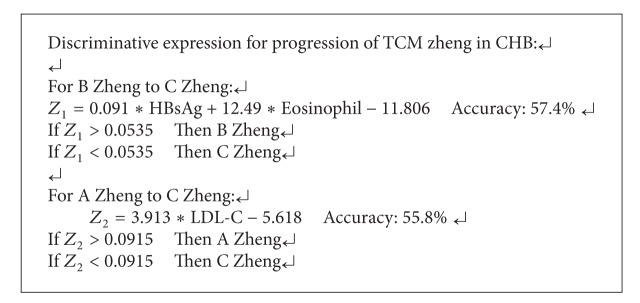
Critical lab indicators of TCM zheng progression in CHB.

**Table 1:** The selected attributes from CFS-GA algorithm.

Selected lab indicators from CFS-GA			
(1) TBIL	(7) IgG	(13) Alpha 1 globulin	(19) Eosinophil percentage
(2) TT	(8) Cr	(14) Beta globulin	(20) Hemoglobin
(3) HBsAg	(9) Blood glucose	(15) Gamma globulin	(21) MCV
(4) HBcAb-IgM	(10) TG	(16) Basophil	(22) RBC
(5) PH value	(11) LDL-C	(17) Basophil percentage	
(6) Urobilinogen	(12) Albumin	(18) Eosinophil	

**Table 2:** Decisive lab indicators through C5.0 boost decision tree.

Clinical indicators of C5.0 boost decision tree induction				
(1) HBsAg	(4) PH value	(7) RBC	(10) Eosinophil	(13) TG
(2) LDL-C	(5) Blood glucose	(8) HBcAb-IgM	(11) Alpha 1 globulin	
(3) Hemoglobin	(6) Basophil	(9) Beta globulin	(12) TBIL	

**Table 3:** Classification results of the comparison.

Attribute selection	Dimensions	NBTree Accu. (%)	REPTree Accu. (%)	C5.0 boost Accu. (%)
Non-attribute selection	83	48.36	53.27	58.73
CFS	3	55.64	55.45	57.09
CFS-GA	22	55.64	66.18	73.82

**Table 4:** The differences of the critical lab indicators in the zheng progression of CHB.

	HBsAg IU/mL	Eosinophil 10^9^/L	LDL-C mmol/L
A zheng	242.96 ± 3.16	0.10 ± 0.01	3.09 ± 0.08
B zheng	229.07 ± 9.05	0.12 ± 0.01	2.80 ± 0.16
C zheng	244.48 ± 2.78*	0.11 ± 0.01	2.83 ± 0.10**

HBsAg: hepatitis B surficial antigen; LDL-C: low-density lipoprotein cholesterol. The values were expressed as mean ± S. E. M. The mean of HBsAg in C zheng had a significant difference when compared with B zheng with *P < 0.05; the mean of LDL-C in C zheng had a significant difference when compared with A zheng with **P < 0.05, and the means of Eosinophil had no significant difference between B zheng and C zheng (*P* > 0.05).
